# Arthroscopic Resection of Infrapatellar Fat Pad Impingement Syndrome: Long-Term Clinical Results at Minimum 10-Year Follow-Up

**DOI:** 10.3390/medicina61060997

**Published:** 2025-05-28

**Authors:** Young-Cheol Park, Young-Mo Kim, Yong-Bum Joo

**Affiliations:** Department of Orthopedic Surgery, Chungnam National University Hospital, Chungnam National University College of Medicine, Munhwa-dong, Jung-gu, Daejeon 35015, Republic of Korea; ycyeh0830@hanmail.net (Y.-C.P.); longman76@hanmail.net (Y.-B.J.)

**Keywords:** infrapatellar fat pad, arthroscopic, resection, long-term, anterior knee pain, impingement

## Abstract

*Background and Objectives*: Infrapatellar fat pad impingement syndrome (IFPIS) is a relatively underdiagnosed cause of anterior knee pain. While conservative management is the initial approach, some patients require surgical intervention. This study aimed to evaluate the long-term clinical and radiologic outcomes following arthroscopic resection of the infrapatellar fat pad in patients with IFPIS. *Materials and Methods*: Eighteen patients (10 females, 8 males; median age 22) diagnosed with IFPIS and unresponsive to conservative therapy underwent arthroscopic partial or subtotal resection between 2007 and 2013. Diagnosis was based on physical examination (Hoffa’s test), MRI findings, and response to lidocaine injection. Clinical outcomes (VAS, IKDC-2000, Kujala, Lysholm, Tegner activity scores) and radiologic assessments (ISR, CDI, PFJ osteoarthritis grade) were evaluated preoperatively, at 2 years, and at a final follow-up (mean 148.7 months). *Results*: All clinical scores significantly improved postoperatively. VAS decreased from 7.25 ± 0.79 to 2.43 ± 1.50 at 2 years, and to 3.66 ± 1.50 at the final follow-up (*p* < 0.001). Similar long-term improvements were observed in the Kujala, IKDC-2000, Lysholm, and Tegner scores (all *p* < 0.001). Radiographic parameters including ISR and CDI remained stable, and there was no statistically significant progression in patellofemoral osteoarthritis. However, 5 of 18 patients (27.8%) reported persistent symptoms at long-term follow-up. *Conclusions*: Arthroscopic resection of the infrapatellar fat pad in patients with IFPIS showed favorable and sustained clinical outcomes over a 10-year follow-up, without significant radiological changes. These results suggest that arthroscopic resection is a viable treatment option when accurate diagnosis is established.

## 1. Introduction

There are three primary anterior knee fat pads, such as the quadriceps suprapatellar, pre-femoral suprapatellar, and infrapatellar retro-patellar tendon (Hoffa’s fat pad), all of which may experience symptomatic impingement [1,2]. The Hoffa pad, also known as the infrapatellar fat pad (IPFP), is an extra-synovial, intracapsular structure that occupies the majority of the anterior knee compartment. Among many structures, one of the diseases well known to cause AKP is infrapatellar fat pad impingement syndrome [3,4,5,6].

The infrapatellar fat pad (IPFP) is also recognized as a potential contributor to anterior knee pain. In 1904, Hoffa described a condition involving isolated infrapatellar fat pad impingement, characterized by inflammatory enlargement and hyperplasia of adipose tissue with accompanying fibrosis and calcification, thought to result from trauma [7]. Patients with infrapatellar fat pad syndrome (IFPS) typically complain of a sharp or burning pain in the front of the knee, located beneath and adjacent to the patellar tendon near its lower attachment. Symptoms are often aggravated by full knee extension, activities like climbing stairs, or maintaining the knee in a flexed position [8,9,10].

Treatment of infrapatellar fat pad impingement syndrome typically begins with non-surgical interventions, including physiotherapy, taping techniques, and targeted muscle strengthening. In some cases, these measures may be supplemented by injections of local anesthetics and/or corticosteroids to reduce inflammation and pain [6,11,12,13]. When conservative management fails to relieve symptoms sufficiently, various surgical options may be employed to address infrapatellar fat pad pathology. These include partial or subtotal resection of the fat pad, removal of fibrotic tissue, anterior interval decompression, synovial membrane excision, release of the infrapatellar plica, and denervation procedures targeting the lower pole of the patella. Several studies have found that arthroscopic resection of the fat pad can be an effective treatment. In 1991, Magi et al. first reported the good results of arthroscopic resection, and after that, Ogilvie-Harris, Kumar, and Kim et al. reported similar results [6,11,14,15].

The epidemiology of infrapatellar fat pad impingement syndrome is unknown [16]. The low documented incidence could be due to the difficulty in diagnosing the condition and its tendency to be adequately treated with conservative management [17]. Infrapatellar fat pad impingement syndrome is thought to be frequently overlooked in imaging studies as it has been detected in only about 1% of patients undergoing knee arthroscopy [15,17]. Therefore, there have been few recent papers reporting long-term follow-up results after surgical treatment because it is difficult to diagnose infrapatellar fat pad impingement syndrome, and in many cases the diagnosis is made after excluding other diseases. It is not easy to perform surgery after accurate diagnosis and report long-term results.

Therefore, the current study then aims to evaluate the short-term and long-term clinical outcomes of arthroscopic resection for patients with infrapatellar fat pad impingement syndrome. The hypothesis is that arthroscopic resection results in favorable clinical outcomes for patients with infrapatellar fat pad impingement syndrome without radiological changes at a 10-year follow-up.

## 2. Materials and Methods

### 2.1. Patient Selection and Diagnosis

Collection of data in our registry was approved by our Institutional Review Board (No. CNUH 2024-06-014). All patients provided informed consent before participation and their medical records and arthroscopic photographs were analyzed.

We followed a consecutive of 38 patients with infrapatellar fat pad impingement syndrome treated by arthroscopic resection at our institution between March 2007 and April 2013. We diagnosed infrapatellar fat pad impingement syndrome if there was a complaint of sharp pain in the infrapatellar region and two or more of following: (1) a positive Hoffa’s test [6,14,18,19], (2) suspicious impingement of the IPFP between the patella and femoral trochlea evident on magnetic resonance imaging (MRI) in the absence of other intra-articular abnormalities, and (3) pain relief or recovery of range of motion after lidocaine injection into the affected area. Surgery was undertaken in those patients who had consistent symptoms despite more than 3 months of conservative treatment [6]. Finally, only patients with arthroscopically confirmed impingement of the infrapatellar fat pad impingement syndrome were included.

Hoffa’s test was performed by first flexing the patient’s knee, then applying manual pressure to either the medial or lateral side of the patellar tendon. While maintaining this pressure, the knee was passively extended. This maneuver causes the infrapatellar fat pad to be compressed into the patellofemoral joint, potentially provoking pain. The test was considered positive for fat pad impingement if the patient experienced pain or discomfort during the last 10 degrees of knee extension [6,14,18,19].

On MRI, findings suggestive of infrapatellar fat pad impingement include the following: (1) infrapatellar fat pad hypertrophies and becomes impinged between the femur and tibia, (2) fibrotic thickening within the infrapatellar fat pad, and (3) edema and inflammatory change [20,21]. In this study, the presence of any one of the three findings was considered a positive MRI finding.

We excluded patients with the following criteria: (1) history of previous knee surgery, (2) meniscal or ligamentous lesions requiring concomitant treatment, (3) inflammatory arthritis, (4) space-occupying knee lesion, (5) psychiatric problems, and (6) no radiological examination at the outpatient follow-up period of 2 years after surgery. A total of 18 patients ultimately met the inclusion criteria and were included in the final analysis.

**Figure 1:**
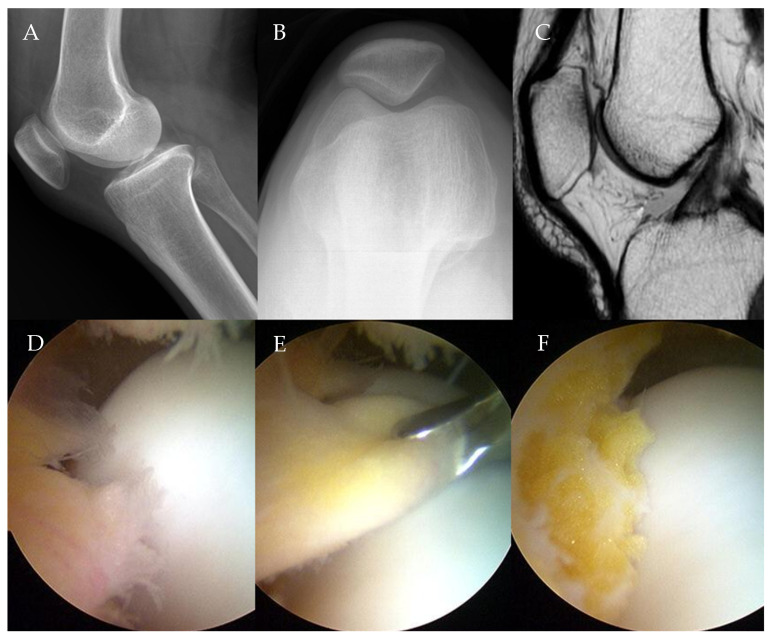
A 46-year-old female patient complaining of anterior knee pain of VAS 6 points that did not improve despite conservative treatment for more than 6 months. (**A**) Lateral knee radiograph showing an Insall–Salvati ratio of 0.98. (**B**) Merchant view showing K-L grade 0. (**C**) MRI showing infrapatellar fat pad volume of 656.24 mm^2^ and inflammatory and edematous changes. (**D**) Arthroscopic finding showing infrapatellar fat pad impingement. (**E**) Resection of the infrapatellar fat pad with a basket. (**F**) Infrapatellar fat pad after arthroscopic subtotal resection.

Of the 18 patients, 11 received partial and 7 underwent subtotal arthroscopic resection of the infrapatellar fat pad due to impingement syndrome (Figure 1).

The extent of fat pad resection was categorized as either partial or subtotal. Partial resection involved the removal of only the impinged portion of the infrapatellar fat pad, whereas subtotal resection entailed excising approximately two-thirds of the tissue, leaving the basal third intact [6]. The amount of resection was based on the fact that the infrapatellar fat pad did not impinge upon knee motion in arthroscopic findings [6].

### 2.2. Surgical Technique

All surgeries were performed by one experienced surgeon (K.Y.M). All patients underwent surgery using the same method, and partial or subtotal resection was performed depending on the volume of impingement of the infrapatellar fat pad [6]. All surgeries were performed under general anesthesia using a pneumatic tourniquet. We used the anterolateral (AL) or superolateral (SL) portal as a viewing portal and the anteromedial (AM) or SL portal as a working portal. The 30° arthroscope was inserted through the AL portal and a routine arthroscopic examination of the whole knee joint was performed. Intraarticular lesions were explored using careful probing through the anteromedial (AM) portal. Subsequently, the arthroscope was shifted to the superolateral (SL) portal to evaluate fat pad impingement between the patella and femoral trochlea. Importantly, with the knee in full extension, the infrapatellar fat pad was observed to be compressed between these two structures. The impinged tissue was then excised using a motorized shaver introduced through the AM portal. While looking through the AL portal, the shaver was placed through the SL portal. After partial or subtotal resection of the IPFP, we checked if the fat pad would be impinged during range of motion of the knee. A tissue specimen from the affected part of the infrapatellar fat pad was sent for histologic study. Additional pathologies were meticulously searched and excluded. From the first postoperative day, range of motion exercises using a continuous passive motion device were started, and quadriceps muscle strengthening was performed several times daily. Full weight-bearing was allowed from the first postoperative day, but the status of weight-bearing was adjusted according to the patient’s individual condition.

### 2.3. Clinical Evaluation

Clinical data were obtained preoperatively, at the two-year postoperative interval and at the final follow-up. Although outpatient monitoring extended beyond two years, only the two-year and final follow-up data were included in the analysis for this study. Five clinical outcome parameters were evaluated: the 10-point visual analog scale (VAS) for pain (0 indicating no pain, 5 for moderate pain, and 10 for the most severe pain), the subjective International Knee Documentation Committee (IKDC) 2000 score, the Lysholm and Kujala knee scores, and the Tegner activity scale. Clinical evaluations were conducted by an author (J.Y.B) who was not involved in the surgical procedures or radiographic analyses. Postoperative complications were assessed both immediately after surgery and at the final follow-up, including potential issues such as neurovascular injury, infection, patellar tendon damage, and alterations in patellar height.

### 2.4. Radiologic Measurements

**Figure 2:**
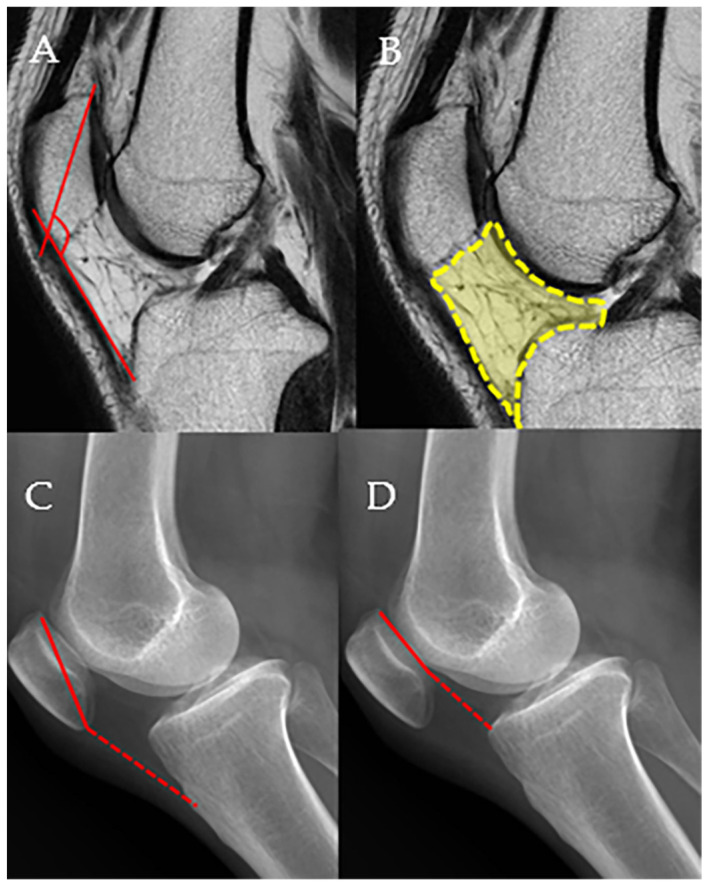
The measurement methods for the patella–patellar tendon angle (PPTA), infrapatellar fat pad volume (IPFV), Insall–Salvati ratio (ISR), and Caton–Deschamps index (CDI) are described. The procedures for PPTA and IPFV measurements are detailed in the main text. (**A**) PPTA, (**B**) IPFV, (**C**) ISR, the ratio of the dashed line to the solid line, (**D**). CDI, the ratio of the dashed line to the solid line.

Before surgery and during follow-up, MRI, weight-bearing whole leg AP radiograph, standing knee antero-posterior (AP), standing knee postero-anterior 45°, knee lateral views of radiographic images, and non-weight-bearing Merchant view of patellofemoral joint were obtained. Magnetic resonance imaging was conducted using either 1.5-Tesla or 3.0-Tesla high-field superconducting scanners. All patients underwent MRI without a contrast agent with the knee in full extension while in the supine position. All MRI and plain radiographic measurements were made on a picture archiving and communication system workstation (Maroview, version 5.4.10.52; Marotech, Seoul, Republic of Korea) by two of the authors (P.Y.C, K.Y.M), who are both experienced orthopedists. Measurements were performed twice by each of the two authors. The patella–patellar tendon angle (PPTA) and infrapatellar fat pad volume (IPFV) were measured on MRI [3], and Insall–Salvati ratio (ISR), Caton–Deschamp index (CDI), and the degree of arthritis in patella-femoral compartment were measured on simple radiographs [22,23] (Figure 2). The PPTA was evaluated on T1-weighted midsagittal images to analyze the sagittal patellar alignment of the patella-femoral joint (PFJ). The PPTA was defined as the angle between the line joining the upper and lower patellar poles and the line through the mid-portion of the patellar tendon [3]. The infrapatellar fat pad (IPFP) lies within the joint capsule but outside the synovial lining, bordered anteriorly by the patellar tendon and posteriorly by the synovial membrane of the femorotibial joint. Superiorly, it connects to the inferior pole of the patella and extends posteriorly toward the intercondylar notch through two alar folds, which merge to form the infrapatellar plica. It is situated adjacent to the femoral trochlear cartilage in the superoposterior region. To calculate its area, the IPFP was outlined manually on sagittal T2-weighted MRI slices at the level showing the greatest cross-sectional dimension [3]. The Insall–Salvati index was calculated as the ratio of the length of the longitudinal patellar diameter to the length of the line connecting the most inferior point of the patella to the patellar insertion site on the tibial tuberosity [22]. In the sagittal plane, the Caton–Deschamps index was calculated as the ratio of the length of the retropatellar surface to the length of the line connecting the most inferior point of the patella to the retropatellar surface of the tibial plateau [22].

On the date of the last follow-up, the patella-femoral joint was also evaluated on non-weight-bearing Merchant view. The Merchant view images were taken with the knee flexed to 45°, and the angle of flexion was standardized using a goniometer. All radiologic measurements were made with the Merchant view standardized to the same angle. Radiographic assessment of the knee was performed to determine the severity of patella-femoral osteoarthritis utilizing the K-L grading system [23]. Merchant view radiographs at baseline and the 2-year postoperative data were evaluated with arthritis graded according to the following scale: none (grade 0), doubtful (grade 1), minimal (grade 2), moderat(grade 3), or severe (grade 4). To minimize potential recall bias, measurements were conducted two weeks apart. Each author performed the assessments independently, and no discussion of the results took place. Furthermore, all evaluations were carried out in a blinded manner, without access to patient information.

### 2.5. Statistical Analysis

Statistical analysis was performed using SPSS version 26.0 (IBM Co., Armonk, NY, USA) with a significance set at *p* < 0.05. Continuous variables were reported as the mean ± standard deviation, with nonparametric variables reported as medians with ranges. Testing of normality and variance homogeneity was performed with the Kolmogorov–Smirnov and Levene tests. Paired *t* test and Wilcoxon signed-rank test were used to compare baseline and final patient-reported outcomes. All statistical tests were 2-sided, with *p* values < 0.05 considered statistically significant. To measure inter- and intraobserver agreement, the intraclass correlation coefficient (ICC) for the radiologic measurements was assessed. Sample size calculation was performed using G*Power version 3.1.9.2. To calculate the sample size, the alpha and effect size f values were used. Statistical power was set at 0.80, the alpha level was 0.05, and effect size f was 0.40, respectively. The required sample size was 18 for infrapatellar fat pad impingement group.

## 3. Results

A total of 18 patients were enrolled. In-person follow-up visits were substituted with phone interviews for all patients who were not able to arrange a clinic visit at final follow up. The preoperative demographic and preoperative data are summarized in Table 1, postoperative clinical results are summarized in Table 2, and postoperative radiographic results summarized in Table 3.

This included ten female (55.6%) and eight male (44.4%) patients. The median age of the patients was 22 years (range 15–48 years). The median duration of follow-up was 148.7 months (range 122.4–154.0 months). There were no adverse events associated with arthroscopic surgery noted at any time during the follow-up period.

An amount of 15 of 18 (83.3%) patients showed Hoffa’s test positive findings, and MRI was performed in all patients, but positive findings were obtained in 11 (61.1%) patients.

All patients underwent histologic study, and the results were consistent with Hoffa’s disease in 3 out of 18 (16.7%) patients, synovial hyperplasia in 9 out of 18 (50.0%) patients, and mild fibrosis in 5 out of 18 (27.8%) patients.

Postoperative data indicated statistically significant changes in patient-reported outcomes. The VAS score decreased from a preoperative value of 7.25 ± 0.79 to 2.43 ± 1.50 at two years following surgery. At the final follow-up, the VAS score slightly increased to 3.66 ± 1.50, but remained significantly lower compared to the preoperative value (*p* < 0.001). The Kujala score improved from 52.35 ± 5.23 preoperatively to 88.61 ± 5.53 at two years, and was maintained at 82.36 ± 6.53 at the final follow-up (*p* < 0.001). The IKDC 2000 score increased from 48.50 ± 8.38 before surgery to 82.39 ± 6.25 at two years, followed by a slight decrease to 77.49 ± 5.19 at the final evaluation (*p* < 0.001). The Tegner activity scale rose from a baseline of 3.0 ± 1.0 to 6.01 ± 0.87 postoperatively at two years, and was slightly reduced to 5.83 ± 0.76 at the last follow-up (*p* < 0.001). Lysholm score also showed improvement, rising from 49.10 ± 6.54 preoperatively to 87.92 ± 5.81 at two years, and slightly decreasing to 80.12 ± 5.77 at the final follow-up (*p* < 0.001) (Table 2).

Radiographic assessments showed no statistically significant differences in patellar height. The Insall–Salvati ratio (ISR) remained unchanged, with a mean value of 1.04 ± 0.18 before surgery and 1.04 ± 0.19 two years after surgery (*p* > 0.05). Similarly, the Caton–Deschamps index (CDI) showed no significant change, measured at 1.05 ± 0.14 preoperatively and 1.07 ± 0.17 postoperatively (*p* > 0.05). However, 2 out of 18 (11.1%) had checked ISR values of 1.22 and 1.63, respectively, indicating patella alta (Table 3).

Evaluation of patellofemoral joint status based on arthritis grading revealed a minor shift in distribution. Preoperative grading showed 14 patients with grade 0, four with grade 1, and none with grades 2 to 4. At two years postoperatively, the distribution changed to 14 with grade 0, two with grade 1, and two with grade 2, with no patients in grades 3 or 4. This change did not reach statistical significance (*p* > 0.05), indicating no significant progression in patellofemoral joint degeneration over the observed period (Table 3).

A total of 5 out of 18 (27.8%) patients complained of pain similar to before surgery at the last follow-up.

There were no statistically significant differences in clinical and radiologic outcomes between the partial resection group (*n* = 11) and the subtotal resection group (*n* = 7).

The interobserver reliability was high for all measurements (ICC > 0.90), as was the intraobserver reliability (ICC > 0.90).

## 4. Discussion

The most important finding of this study was that the arthroscopic resection for patients with infrapatellar fat pad impingement syndrome improved clinical outcomes significantly without radiographic change at the long-term follow-up of mean 148.7 months.

In this study, arthroscopic resection of an infrapatellar fat pad showed statistically significant results in VAS, Kujala, IKDC-2000, Lysholm, and Tegner activity score at 2 years after surgery and at the final follow-up period of more than 10 years after surgery. In total, 13 out of 18 patients showed satisfactory results, indicating their previous sports activity level. This result is considered to be similar to that of previous research. Previous studies have demonstrated that arthroscopic excision of the infrapatellar fat pad can be an effective treatment option for managing impingement. For instance, Kumar et al. reported a significant improvement in knee function following infrapatellar fat pad resection, with Lysholm scores increasing from 37.4 before surgery to 90.8 at final follow-up. They concluded that targeted arthroscopic removal of the impinging tissue provided sustained symptom relief with minimal complications or morbidity [6,14]. Ogilvie-Harris and Giddins also reported marked improvement in both symptoms and joint function after resection of the fat pad at a mean follow-up of 76 months [6,15]. Although this study reported similar results to previous studies, its strengths include reporting long-term results of 148.7 months and describing radiologic evaluation.

However, in this study, 5 out of 18 patients complained of persistent pain at a similar level to before surgery during the last follow-up period, which may have several causes.

First, there is a possibility that an accurate diagnosis of infrapatellar fat pad impingement syndrome was not made at the time of surgery. In this study, we attempted to make a comprehensive diagnosis through physical examination including Hoffa’s test, MRI findings, patellofemoral joint evaluation, and histologic evaluation, but the possibility of misdiagnosis cannot be ruled out. Second, it is possible that the diagnosis was made accurately but the exact fat pad volume causing the infrapatellar fat pad impingement was not removed. There have been no reports to date on how much of the infrapatellar fat pad should be excised. Kim et al. compared arthroscopic partial and subtotal resection for infrapatellar fat pad impingement and found that both techniques significantly improved pain and functional scores. No significant differences were observed between the two groups in clinical outcomes after a minimum two-year follow-up [6]. In this study, we believe that the reason for these results was that the volume of fat pat excision was not accurately calculated for each patient.

In the current study, patellar height was evaluated using the Insall–Salvati ratio and the Caton–Deschamps index, but there was no significant difference between preoperative and postoperative 2 years. In particular, mean ISR and CDI were evaluated as normal ranges, which is somewhat contrary to previous studies. Many studies reporting infrapatellar fat pad impingement syndrome mention patella alta as a predisposing factor, but in this study, 2 out of 18 patients (11.1%) had patella alta. A high ISR denotes a high-riding patella (patella alta). High patellar height, TT-TG distance, and trochlear angle have been reported to be predisposing factors for infrapatellar fat pad impingement [24,25,26]. Kim et al. reported that the Insall–Salvati ratio (ISR) was 1.1 in patients with superolateral Hoffa’s fat pad edema, which was significantly higher than the control group (0.9) [24,27]. Similarly, in a cohort of 133 patients with patellofemoral pain, van Middelkoop et al. found Hoffa synovitis in 81 cases. Their analysis revealed a positive association between an increased Insall–Salvati ratio and the presence of synovitis in the infrapatellar fat pad [24,26]. In this study, only two patients showed patellar alta in the patellar height measured before surgery, so the sample size was small for comparison with previous studies, but it seems that there may be a correlation. In addition, in the evaluation of patellar height at the 2-year follow-up, the ISR was 1.04 ± 0.18 before surgery and 1.04 ± 0.19 at 2 years, which showed no significant difference. Therefore, we believe that further research on patellar height and infrapatellar impingement is needed. Hoffa’s fat pad is an intra-capsular structure located inferior to the patella. A high-riding patella may apply traction on Hoffa’s fat pad, leading to edema and fibrotic changes in the structure. These findings suggest that considering patella alta correction may be beneficial in the management of infrapatellar fat pad syndrome. The previously reported literature reported the relationship between patellar maltracking and suprapatellar Hoffa’s fat pad, so it is judged that there may be some differences in the interpretation of the results of this study.

There are many reports on the role of IFP in knee osteoarthritis. In their MRI-based analysis of 174 patients, Cai et al. found that individuals with a larger infrapatellar fat pad volume tended to exhibit increased cartilage volume and fewer structural abnormalities in the knee joint, indicating a potentially protective effect of the IPFP in the context of osteoarthritis [28]. Cuckpaiwong et al. compared fat pad volume at 3, 6, and 12 months using 3T knee MRI, dividing 15 control groups and knee osteoarthritis groups, respectively. Compared to the control group, it was reported that the fat pad volume increased significantly at 3, 6, and 12 months in the knee osteoarthritis group [29]. This result does not seem to be able to completely rule out the possibility that even in the patients who were defined as failures in the present study, the resected fat pad volume increased again over time and caused impingement.

However, there is also the literature that reported the importance of the alteration of signal intensity of the infrapatellar fat pad rather than infrapatellar fat pad volume. He et al. reported a smaller value of 689.7 ± 129.3 mm^2^ in the healthy group and 606.8 ± 92.5 mm^2^ in OA patients, suggesting that the metabolism-related function of IPFP, which can be reflected by the IPFP signal, might play a more critical role in OA progression than its structural function [30]. Although many studies have been conducted on the relationship between infrapatellar fat pad and osteoarthritis, a larger cohort study will be needed to identify the relationship between infrapatellar fat pad and osteoarthritis. In the current study, it was difficult to find significant arthritic changes in patients at the time of diagnosis.

The infrapatellar fat pad (IPFP) can be a cause of AKP, from two different pathologies, Hoffa’s disease [7] or benign tumors [20]. Surgical excision followed by histological analysis has commonly been used to manage such tumorous lesions and confirm the diagnosis [17]. Despite its clinical relevance, the existing literature on the surgical treatment of infrapatellar fat pad (IPFP) disorders remains limited, primarily consisting of isolated case reports and small case series [17]. Kumar et al. performed histological anastomosis in 34 patients at high portal arthroscopic resection [14]. A total of three histological classifications were made, with type I being an acute lesion, type 2 being a chronic lesion without fibrosis, and type 3 being a chronic lesion with fibrosis. There were 10, 14, and 10 patients in each type, respectively, and there were no cases of benign tumorous condition [14]. In addition, Park et al. diagnosed infrapatellar fat pad impingement syndrome in a 70-year-old woman, performed arthroscopic fat pad resection, and performed a biopsy [31]. The findings of adipocyte necrosis, dystrophic calcification, and adipocytes surrounded by inflammatory cells were consistent with the preoperative MRI, and this finding supported Hoffa’s disease [31].

While trauma and degenerative joint disease are the most frequent causes of fat pad abnormalities, less common etiologies such as inflammatory or neoplastic conditions may also occur. Tumorous involvement of the fat pad can present either as a localized (solitary) lesion or as diffuse diseases [32]. In this study, a biopsy was performed on all patients after surgery, and no tumorous condition was observed in any patient. However, it is considered absolutely necessary to exclude other diseases such as pigmented villonodular synovitis (PVNS), ganglion cyst, and hemangioma through thorough radiologic evaluation before surgery and biopsy after surgery.

This study has several limitations. First, due to its retrospective design and lack of randomization, there is a potential risk of selection bias. Moreover, the absence of a control group treated with conservative methods limits the ability to draw comparative conclusions. Second, the sample size was small. A prospective study with a large sample size is required. Third, although this study reported long-term results at a minimum of 10 years, the long-term results clearly showed clinical outcomes. During the last follow-up period, radiologic evaluation (including MRI) and second-look arthroscopy were not available for all patients, and clinical results depended only on patient-reported outcome data. Hence, the status of the resected infrapatellar fat pad could not be evaluated at the final follow-up period. Lastly, this study did not include an analysis of the potential effects of partial versus subtotal resection of the infrapatellar fat pad on the patellar tendon or other anatomical structures within the knee joint.

## 5. Conclusions

In conclusion, after an arthroscopic resection for patients with infrapatellar fat pad impingement syndrome, favorable clinical outcomes were shown about 72.2% of the patients during a minimum follow-up of 148.7 months. Hence, if an accurate diagnosis is a prerequisite, arthroscopic resection is worth considering as a treatment option.

## Figures and Tables

**Table 1:** Summary of patient’s demographics data, preoperative-clinical, radiologic, and postoperative-histologic results.

Case	Age	Sex	Site	BMI	Follow-Up (Months)	Tegner Activity	Hoffa’s Test	MRI	VAS	PPTA	IFPV	ISR	CDI	PFJ Arthritis (0/1/2/3/4)	Biopsy
1	15	F	R	21.62	148.4	3	positive	positive	7	133.6	528.6	0.92	0.96	0	Consistent with Hoffa’s disease
2	29	M	R	26.18	149.0	5	positive	positive	6	125.3	783.9	1.12	1.31	0	Consistent with Hoffa’s disease
3	46	F	R	23.34	141.8	3	positive	negative	6	133.4	491.4	0.99	0.76	1	Mild synovial hyperplasia
4	21	M	R	22.43	145.2	4	negative	negative	7	144.8	829.0	1.22	1.14	0	Mild synovial hyperplasia
5	19	M	L	21.08	147.1	4	positive	positive	8	136.7	799.8	1.07	1.15	0	Mild fibrosis
6	30	F	L	20.05	135.5	3	positive	negative	7	133.7	626.2	1.63	1.13	0	Synovial hyperplasia and proliferation adipocyte
7	27	M	R	23.45	154.0	4	negative	positive	7	141.1	740.2	1.00	1.00	0	Mild fibrosis
8	47	F	L	21.07	147.5	3	positive	negative	6	130.2	591.2	1.12	0.91	1	Mild fibrosis
9	45	F	L	19.6	132.1	3	positive	negative	8	135.8	627.1	0.81	1.01	0	Increase in mature adipose tissue, consistent with fat pad
10	43	F	R	19.86	137.8	3	positive	negative	6	142.5	530.2	0.88	0.99	0	Mild synovial hyperplasia
11	19	M	L	22.05	145.7	4	positive	positive	7	136.3	777.1	0.87	0.81	1	Mild fibrosis
12	33	F	R	28.23	145.3	2	positive	negative	8	143.0	585.9	1.03	1.14	0	Mild fibrosis
13	22	M	R	24.81	148.5	3	positive	negative	8	138.4	886.0	1.12	1.09	0	Consistent with Hoffa’s disease
14	48	M	L	29.21	122.5	4	positive	positive	8	137.0	586.3	0.92	0.81	1	Mild synovial hyperplasia
15	28	F	L	21.04	141.1	2	positive	negative	7	134.2	657.3	0.81	1.00	0	Mild synovial hyperplasia with fatty change
16	20	F	L	19.77	122.4	2	positive	positive	8	130.0	511.8	1.13	1.00	0	Mild synovial hyperplasia
17	30	F	L	21.51	149.8	2	positive	negative	7	139.7	637.4	0.96	1.12	0	Mild synovial hyperplasia with fatty change
18	23	M	L	23.63	149.5	4	negative	negative	8	136.3	769.4	1.12	1.18	0	Synovial hyperplasia and increased capillaries

BMI, body mass index; VAS, visual analog scale score; PPTA, patella–patellar tendon angle; IFPV, infrapatellar fat pad volume; ISR, Insall–Salvati ratio; CDI, Caton–Deschamps index.

**Table 2:** Comparison of preoperative, 2-year postoperative, and final follow-up patient-reported outcomes.

Variables	Preoperative	Postoperative (2 Years)	Postoperative (Final Follow-Up)	*p*-Value ^a^	*p*-Value ^b^
VAS	7.25 ± 0.79	2.43 ± 1.50	3.66 ± 1.50	<0.001	<0.001
Kujala	52.35 ± 5.23	88.61 ± 5.53	82.36 ± 6.53	<0.001	<0.001
IKDC 2000	48.50 ± 8.38	82.39 ± 6.25	77.49 ± 5.19	<0.001	<0.001
Tegner activity	3.0 ± 1.0	6.01 ± 0.87	5.83 ± 0.76	<0.001	<0.001
Lysholm	49.13 ± 7.12	83.17 ± 6.54	76.83 ± 5.72	<0.001	<0.001

Values are presented as mean ± standard deviation; VAS: visual analog scale score; WOMAC: Western Ontario and McMaster Universities Osteoarthritis index; IKDC: International Knee Documentation Committee; ^a^:comparison preoperative and 2 years postoperative; ^b^: comparison preoperative and final follow-up postoperative.

**Table 3:** Comparison of preoperative and 2-year postoperative radiographic results.

Variables	Preoperative	Postoperative (2 Years)	*p*-Value
ISR	1.04 ± 0.18	1.04 ± 0.19	n.s
CDI	1.05 ± 0.14	1.07 ± 0.17	n.s
PF arthritis(0/1/2/3/4)	14/4/0/0/0/	14/2/2/0/0/	n.s

Values are presented as mean ± standard deviation; ISR: Insall–Salvati ratio; CDI: Caton–Deschamps index; n.s: no significant difference.

## Data Availability

The data presented in this study are available on request from the corresponding author.

## Abbreviations

The following abbreviations are used in this manuscript:

AKP	Anterior knee pain
PFJ	Patellofemoral joint
IPFP	Infrapatellar fat pad
MRI	Magnetic resonance imaging
AL	Anterolateral
SL	Superolateral
AM	Anteromedial
VAS	Visual analog scale
IKDC	International Knee Documentation Committee
AP	Anteroposterior
PPTA	Patella–patellar tendon angle
IPFV	Infrapatellar fat pad volume
ISR	Insall–Salvati ratio
CDI	Caton–Deschamp index
ICC	Intraclass correlation coefficient
OA	Osteoarthritis
PVNS	Pigmented villonodular synovitis
